# Engineering *Escherichia coli* for Ergothioneine Production via Metabolic Engineering and Fermentation Optimization

**DOI:** 10.3390/microorganisms14051088

**Published:** 2026-05-11

**Authors:** Yuyang Liu, Yaxin Wen, Ruizheng Hu, Ruyue Han, Dong Liu, Hailing Zhang

**Affiliations:** College of Life Sciences, Yantai University, 30 Qingquan Road, Yantai 264005, China

**Keywords:** ergothioneine, heterologous biosynthesis, *Escherichia coli*, protein expression, metabolic engineering, fermentation engineering

## Abstract

Ergothioneine (EGT), a naturally occurring amino acid derivative with potent antioxidant and cytoprotective properties, is widely applied in the food, cosmetic, and medical industries. Traditional production methods are limited by high costs, low efficiency, and environmental concerns, so microbial fermentation serves as a sustainable alternative for EGT production. In this study, *Escherichia coli* BL21 (DE3) was employed as the chassis strain. First, a basic EGT-producing engineered strain was constructed by heterologously expressing the *egtB* gene from *Methylobacterium pseudosasicola* along with the *egtD* and *egtE* genes from *Mycobacterium smegmatis*. This initial strain achieved a yield of 84.84 ± 1.64 mg/L of EGT in shake-flask cultures. To enhance production, solubility-enhancing tags were introduced to improve the soluble expression of the key enzymes, and metabolic pathways were rationally engineered to strengthen the supply of essential precursor amino acids. These modifications led to the development of a high-yield EGT strain. After optimizing the fermentation process, the best results were achieved using a medium with glycerol as the carbon source, 0.5 g/L of histidine, 1.5 g/L of methionine, and 1.0 g/L of cysteine, along with induction at 25 °C using 0.2 mM IPTG for 120 h. Under these conditions, the final EGT yield reached 385.70 ± 4.86 mg/L. The engineered strain for EGT synthesis and optimized fermentation strategy developed in this study offer a useful basis for further process development.

## 1. Introduction

Ergothioneine (EGT) is a natural antioxidant and sulfur-containing amino acid derived from histidine. It is the only known naturally occurring 2-thioimidazole amino acid, first discovered and isolated from *Claviceps purpurea* in rye by Charles Tanret in 1909 [1]. EGT exists as two tautomers: the thione and thiol forms. At physiological pH, EGT exists predominantly in the thione form because the thione group is more stable than the sulfhydryl group [2]. Compared to other biological thiols such as glutathione and lipoic acid, EGT, which has a higher standard redox potential, exhibits lower reactivity, enhanced antioxidant capacity, and superior chemical and thermal stability [3,4].

Due to its remarkable antioxidant activity, EGT has been widely applied in the food, cosmetic, and medical industries [5,6]. In the food sector, EGT can effectively delay oxidative deterioration by inhibiting lipid peroxidation, demonstrating significant value in color stabilization and preservation [7,8,9,10,11,12]. In the cosmetic industry, EGT provides benefits such as anti-aging, skin brightening, and defense against ultraviolet (UV)-induced damage [13,14,15,16]. Furthermore, in medical and nutraceutical fields, EGT has shown substantial therapeutic and protective potential against neurological disorders, cardiovascular diseases, reproductive dysfunction, and other related diseases [17,18,19,20,21,22].

The preparation methods for ergothioneine primarily consist of three categories: natural extraction, chemical synthesis, and biosynthesis [23,24]. Natural extraction utilizes raw materials such as edible fungi and fish to obtain EGT through methods like solvent extraction; however, this approach is limited by low raw material utilization, poor extraction efficiency, and insufficient product purity [25,26,27,28]. Chemical synthesis mainly includes chiral catalysis based on 1,3-dihydroxyacetone [5] and thiol group introduction based on 2-mercaptoimidazole [29]. Nevertheless, these methods face challenges such as high costs, complex procedures, and environmental pollution [30]. In contrast, microbial synthesis has garnered significant attention due to its economic viability and environmental friendliness. On one hand, natural EGT-accumulating microorganisms are employed as production hosts, such as *Mycobacterium smegmatis* [31], *Methylobacterium* [32], *Rhodotorula glutinis* [33], *Cyanobacteria* [34], and *Chlorobium limicola* [35]. On the other hand, well-characterized chassis strains, including *Escherichia coli* [36], *Corynebacterium glutamicum* [37], *Bacillus licheniformis* [38], *Saccharomyces cerevisiae* [39], *Rhodosporidium toruloides* [40], *Yarrowia lipolytica* [41], *Kluyveromyces lactis* [42], and *Aspergillus oryzae* [43], are utilized for heterologous expression of EGT biosynthetic genes.

To date, four core biosynthetic pathways have been clearly identified. Among bacteria, Seebeck et al. [31] first discovered the actinobacterial pathway in 2010, identifying the EGT biosynthetic gene cluster, *egtABCDE*, in *Mycobacterium smegmatis*. This cluster encodes five enzymes that facilitate EGT synthesis through five distinct catalytic steps. Initially, histidine undergoes methylation to form hercynine (HER). Subsequently, HER reacts with γ-glutamylcysteine (γ-GC) to produce γ-glutamyl-hercynylcysteine sulfoxide (γ-GC-HER). This intermediate is then hydrolyzed to yield hercynylcysteine sulfoxide (Cys-HER). Finally, Cys-HER undergoes cleavage to generate ergothioneine. For example, Tanaka et al. [44] overexpressed the *egtABCDE* gene cluster derived from *Mycobacterium smegmatis*, along with mutant *cysE*, mutant *serA*, and the *ydeD* gene, and knocked out the *metJ* gene in *Escherichia coli* (*E. coli*). By combining these modifications with a fed-batch fermentation strategy, they achieved an EGT yield of 1.31 g/L in a 3 L fermenter after 216 h. Recent studies have revealed that EgtB in *Methylobacterium strains* possesses a unique functional property: it can directly utilize cysteine as the sulfur donor to catalyze the conversion of HER to Cys-HER. Consequently, the ergothioneine biosynthetic pathway requires only three enzymes—EgtB, EgtD, and EgtE—thereby streamlining the process from five steps to three [45,46]. Kamide et al. [47] overexpressed *egtB* from *Methylobacterium* and *egtD* and *egtE* from *M. smegmatis* in an *E. coli* strain engineered for high cysteine production and enhanced S-adenosylmethionine (SAM) supply; an EGT yield of 657 mg/L was achieved after 192 h of shake-flask fermentation. In another study, Burn et al. [48] identified EanA and EanB from the anaerobic bacterium *Chlorobium limicola*. EanA catalyzes the conversion of histidine to HER, after which EanB transfers a sulfur atom to the imidazole ring of HER under anaerobic conditions to generate ergothioneine. Liu et al. [38] overexpressed the *EanA* and *EanB* genes from *Chlorobium limicola*, along with screened homologous genes *EanAN* and *EanBN*, in *Bacillus licheniformis.* By optimizing culture conditions, they obtained an EGT yield of 643.8 ± 135 mg/L after 140 h of shake-flask fermentation. In fungi, Hu et al. [49] identified Egt1 and Egt2 from *Neurospora crassa*. Egt1 directly catalyzes the synthesis of Cys-HER, which is subsequently converted into ergothioneine via Egt2 catalysis. Chen et al. [42] integrated *Shegt1* and *Shegt2*, derived from *Stereum hirsutum*, into the genome of *Kluyveromyces lactis*. Combined with a fed-batch fermentation strategy, an EGT yield of 398.64 mg/L was achieved in a 5 L fermenter after 96 h.

Currently, the microbial strains engineered for ergothioneine biosynthesis still show considerable room for yield improvement. For instance, the introduction of a heterologous biosynthetic pathway imposes an increased metabolic burden on host cells and consequently impairs cell growth; meanwhile, intracellular accumulation of the product also elevates the difficulty and cost of downstream separation and purification. To address these existing challenges, *E. coli* BL21 (DE3) was employed as the chassis cell in this study. An engineered EGT-producing strain was successfully constructed by heterologously expressing the *egtB* gene from *Methylobacterium pseudosasicola* along with the *egtD* and *egtE* genes from *Mycobacterium smegmatis*. On this basis, solubility-enhancing tags were introduced to improve the soluble expression levels of key enzymes. Furthermore, the supply capacity of three critical precursors, namely histidine (His), methionine (Met), and cysteine (Cys), was strengthened through metabolic engineering strategies. By systematically optimizing the fermentation process, the efficient synthesis of EGT was eventually achieved, providing a useful basis for further process development of ergothioneine.

## 2. Materials and Methods

### 2.1. Plasmids and Strains

*E. coli* BL21 (DE3), *E. coli* DH5α, and the plasmids pRSFDuet-1 and pCDFDuet-1 were maintained in our laboratory. The antibiotics were used at the following final concentrations: kanamycin (Kan), 50 μg/mL; and streptomycin (Sm), 50 μg/mL. *E. coli* BL21 (DE3) was utilized as the host strain for ergothioneine biosynthesis. The plasmids and strains used in this study are listed (Table 1), while the primer sequences are provided in the Supplementary Materials (Supplementary Table S1).

### 2.2. Reagents

Ergothioneine standard samples were purchased from Shanghai Aladdin Biochemical Technology Co., Ltd. (Shanghai, China). Histidine, methionine, and cysteine were obtained from Beyotime Biotechnology Co., Ltd. (Shanghai, China). Kanamycin, streptomycin, and isopropyl β-D-thiogalactoside (IPTG) were purchased from Beijing Solarbio Science & Technology Co., Ltd. (Beijing, China). All other reagents were of analytical grade and purchased from domestic suppliers.

### 2.3. Media

The ergothioneine fermentation medium was prepared in two separate parts to prevent precipitation. Component A comprised 12.0 g/L tryptone, 24.0 g/L yeast extract, 5.04 g/L glycerol, 2.0 g/L citric acid, and 1.0 g/L MgSO_4_·7H_2_O, while Component B consisted of 2.31 g/L KH_2_PO_4_ and 12.54 g/L K_2_HPO_4_. Both parts were sterilized separately at 121 °C for 20 min. After cooling to room temperature, Component B was aseptically mixed with Component A. Subsequently, the medium was supplemented with 1 mL/L of the trace element solution, which contained 2.4 g/L FeCl_3_·6H_2_O, 0.15 g/L CuCl_2_·2H_2_O, 0.3 g/L ZnCl_2_, 0.8 g/L CoCl_2_·6H_2_O, 1.2 g/L MnSO_4_, 0.075 g/L H_3_BO_3_, 0.3 g/L Na_2_MoO_4_·2H_2_O, and 10.0 g/L CaCl_2_·2H_2_O dissolved in 120 mM HCl. This solution was sterilized by filtration through a 0.22 μm membrane filter. Finally, the pH of the medium was adjusted to 6.8–7.0.

### 2.4. Induction and Fermentation Cultivation

Glycerol stocks stored at −80 °C were streaked onto Luria–Bertani (LB) agar plates containing appropriate antibiotics using a sterile inoculation loop. The plates were incubated inverted overnight at 37 °C. Subsequently, a single colony was picked and inoculated into 5 mL of LB liquid medium with antibiotics and cultivated at 37 °C and 200 rpm for 16 h. The activated seed culture was then transferred at a 1% (*v*/*v*) inoculation rate into 250 mL shake flasks containing 50 mL of ergothioneine fermentation medium [50]. The cultures were grown at 37 °C and 200 rpm; when the OD_600_ reached 0.8–1.0, the induction phase was initiated [51]. Initially, in the stage of engineered strain construction and EGT synthesis verification, the culture medium was supplemented with a baseline formula consisting of amino acids (0.5 g/L His, 1.5 g/L Met, and 0.5 g/L Cys) and cofactors (50 mg/L ferric ammonium citrate and 50 mg/L vitamin B_6_) [52]. Subsequently, to optimize the fermentation process, the effects of induction temperature, IPTG concentration, carbon source types, and supplementation with different amino acids on cell growth and ergothioneine yield were investigated. During this stage, the concentrations of individual amino acids and cofactors remained consistent with the aforementioned culture system. Finally, to determine the optimal supplementation concentrations of the three amino acids, gradients of 0.5, 1.0, and 1.5 g/L were employed to sequentially optimize the supplementation levels of histidine, methionine, and cysteine. Upon completion of the fermentation, cell density was measured using a UV–visible spectrophotometer and indicated by the OD_600_ value [53,54,55], and ergothioneine yield was determined by high-performance liquid chromatography (HPLC) [56]. These measurements were used to evaluate and determine the optimal fermentation conditions.

### 2.5. Construction of Engineering Strains

**Figure 1:**
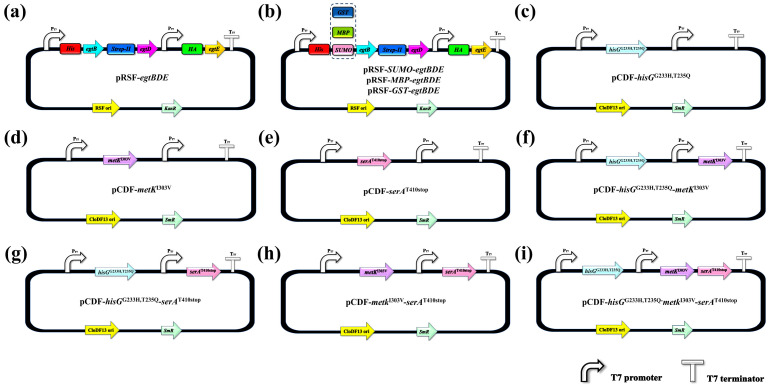
Schematic diagrams of recombinant plasmid construction. (**a**) pRSF-*egtBDE*; (**b**) pRSF-*SUMO-egtBDE*, pRSF-*MBP-egtBDE*, pRSF-*GST-egtBDE*; (**c**) pCDF-*hisG*^G233H,T235Q^; (**d**) pCDF-*metK*^I303V^; (**e**) pCDF-*serA*^T410stop^; (**f**) pCDF-*hisG*^G233H,T235Q^-*metK*^I303V^; (**g**) pCDF-*hisG*^G233H,T235Q^-*serA*^T410stop^; (**h**) pCDF-*metK*^I303V^-*serA*^T410stop^; (**i**) pCDF-*hisG*^G233H,T235Q^-*metK*^I303V^-*serA*^T410stop^.

In this study, the key genes of the EGT biosynthetic pathway, e*gtB*, *egtD*, and *egtE*, were codon-optimized based on the codon preference of *E. coli*. The optimized genes were synthesized by Suzhou Silicon-Based Biotechnology Co., Ltd. (Suzhou, China). The *egtB* gene was derived from *Methylobacterium pseudosasicola*, while *egtD* and *egtE* were obtained from *Mycobacterium smegmatis*. Subsequently, the target genes were sequentially inserted into the high-copy-number vector pRSFDuet-1 using either one-step cloning or restriction-ligation methods [57,58]. Simultaneously, a tag-fusion strategy was employed, where His-tag, Strep-II tag, and HA tag were fused to the N-termini of the three target proteins, respectively, via flexible linkers [59,60,61]. This resulted in the construction of the multi-gene tandem expression plasmid pRSF-*egtBDE* (Figure 1a). After verification by colony PCR, restriction digestion, and DNA sequencing, the recombinant plasmid was transformed into *E. coli* BL21 (DE3) competent cells. The basic engineering strain E2 was obtained after screening on kanamycin-resistant plates and colony PCR verification. Strain E1, harboring the pRSFDuet-1 vector, served as the control. To address the tendency of the key enzyme EgtB to form inclusion bodies during heterologous expression, three solubility-enhancing tags (SUMO, MBP, and GST) [62] were further fused to the N-terminus of the EgtB protein. This led to the construction of the recombinant plasmids pRSF-*SUMO*-*egtBDE*, pRSF-*MBP*-*egtBDE*, and pRSF-*GST*-*egtBDE* (Figure 1b). The verified plasmids were individually transformed into *E. coli* BL21 (DE3) competent cells. Subsequently, the engineering strains E3, E4, and E5 were obtained after screening on kanamycin-resistant plates and verification by colony PCR. To further enhance the supply of precursor substrates (His, Met, and Cys) required for EGT biosynthesis [63,64,65], a series of recombinant plasmids carrying feedback-insensitive mutant genes were constructed using the low-copy-number vector pCDFDuet-1. These included single-mutation plasmids (pCDF-*hisG*^G233H,T235Q^, pCDF-*metK*^I303V^, and pCDF-*serA*^T410stop^) (Figure 1c–e), double-mutation plasmids containing combinations of two mutant genes (pCDF-*hisG*^G233H,T235Q^-*metK^I^*^303V^, pCDF-*hisG*^G233H,T235Q^-*serA*^T410stop^, and pCDF-*metK*^I303V^-*serA*^T410stop^), and a triple-mutation plasmid combining all three mutant genes (pCDF-*hisG*^G233H,T235Q^-*metK*^I303V^-*serA*^T410stop^) (Figure 1f–i). The verified plasmids were individually transformed into the optimal engineering strain E3 obtained from earlier screening. Following selection on dual-antibiotic plates (kanamycin and streptomycin) and verification by colony PCR, engineering strains with single-precursor pathway enhancements (E7, E8, and E9), dual-precursor pathway enhancements (E10, E11, and E12), and the integration of all three precursor pathways (E13) were obtained. Simultaneously, strain E6, harboring the double vectors (pRSFDuet-1 and pCDFDuet-1), served as the control. Ultimately, the systematic construction and optimization of the heterologous EGT biosynthesis strains were accomplished.

### 2.6. Sample Preparation

After 16 h of induction, 10 μL of culture from each group was collected and mounted on glass slides. The bacterial growth at the early induction stage was observed using a phase-contrast microscope at 400× magnification, and representative fields of view were randomly selected and imaged [66]. After 72 h of induction, cultures were harvested by centrifugation. The cells were resuspended in a bacterial protein extraction reagent (Beyotime Biotechnology, Shanghai, China) and incubated at room temperature for 30 min to ensure complete cell lysis. Subsequently, samples of whole-cell protein, the soluble fraction, and the insoluble fraction were prepared separately. At the end of the fermentation, the OD_600_ values of all groups were measured to evaluate bacterial growth. For optimal data accuracy, the measured OD_600_ values were ensured to fall within the range of 0.2 to 0.8. Specifically, 1 mL of the culture was centrifuged, and the supernatant was discarded. The cell pellet was resuspended in ultrapure water to its original volume and appropriately diluted. The absorbance of the diluted suspension at 600 nm was measured using a UV–visible spectrophotometer. Simultaneously, both intracellular and extracellular samples were prepared for analysis. For the extracellular sample, 1 mL of the fermentation broth was centrifuged at 12,000 rpm for 10 min. The supernatant was collected, diluted to fit the standard curve range, and filtered through a 0.22 μm microporous membrane. For the intracellular sample, 1 mL of the fermentation broth was centrifuged at 12,000 rpm for 10 min, and the supernatant was discarded. The cells were resuspended in 1 mL of PBS buffer and lysed using an ultrasonic cell disruptor in an ice bath. The sonication parameters were set as follows: 4 °C, 300 W power, 2 s on, and 4 s off, for a total duration of 10 min. After disruption, the lysate was centrifuged at 12,000 rpm for 10 min, and the resulting supernatant was collected and filtered through a 0.22 μm microporous membrane [67,68].

### 2.7. Establishment of the Ergothioneine Detection Method

The detection conditions were as follows: a Thermo Fisher Scientific Vanquish Series high-performance liquid chromatography (HPLC) system equipped with a UV detector was employed. Separation was performed on a Welch AQ-C18 column (250 mm × 4.6 mm, 5 μm) [69]. The mobile phase consisted of water and acetonitrile at a ratio of 97:3 (*v*/*v*) [70]. The flow rate was set at 0.7 mL/min, and the column temperature was maintained at 30 °C. The detection wavelength was 257 nm, the injection volume was 10 μL, and the total run time was 7 min.

An EGT standard stock solution (500 mg/L) prepared in ultrapure water was serially diluted to obtain standard solutions with concentrations of 5, 25, 50, 100, 200, 400, and 500 mg/L. These solutions were subsequently filtered through a 0.22 μm microporous membrane. The peak areas of EGT at different concentrations were measured at 257 nm using HPLC. A standard curve was then generated by plotting the peak areas (Y) against the corresponding EGT standard concentrations (X). The EGT concentrations in the analytical samples were calculated by substituting their peak areas into this standard curve. To further confirm the product structure, qualitative identification was performed using ultra-performance liquid chromatography–tandem mass spectrometry (UPLC-MS) [71]. The analysis was conducted on a Thermo Scientific Ultimate 3000 system coupled with a Thermo Scientific Q Exactive high-resolution mass spectrometer (Thermo Fisher Scientific, Waltham, MA, USA).

## 3. Results

### 3.1. Protein Expression Analysis of the Engineered Strains

**Figure 2:**
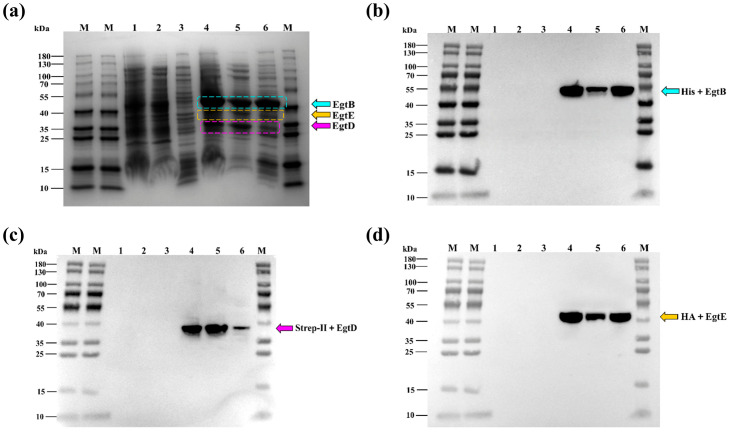
Protein expression and solubility analysis of the recombinant strains. (**a**) SDS-PAGE analysis of the co-expression of EgtB, EgtD, and EgtE. (**b**–**d**) Western blot verification of EgtB (anti-His tag), EgtD (anti-Strep-II tag), and EgtE (anti-HA tag), respectively. Lane M: Protein molecular weight marker; Lane 1: Control strain E1 whole-cell protein; Lane 2: E1 soluble supernatant fraction; Lane 3: E1 insoluble pellet fraction; Lane 4: Engineering strain E2 whole-cell protein; Lane 5: E2 soluble supernatant fraction; Lane 6: E2 insoluble pellet fraction.

After validation by colony PCR (Supplementary Figure S1), restriction enzyme digestion (Supplementary Figure S2), and sequencing (Supplementary Figure S5), the recombinant plasmids were transformed into *E. coli* BL21 (DE3) competent cells to obtain the engineered strain E2. Following induction with IPTG, the protein expression levels were analyzed using SDS-PAGE and Western blot techniques. The theoretical molecular weights of the fusion proteins are provided (Table 2) for reference in identifying the corresponding bands. SDS-PAGE analysis revealed that no distinct bands corresponding to the target proteins were detected in the whole-cell lysate, soluble (supernatant), and insoluble (pellet) fractions of the control strain E1. In contrast, the engineered strain E2 exhibited clear bands of the expected sizes in all corresponding fractions (Figure 2a). Further confirmation was achieved via Western blot analysis, where the target proteins were specifically detected at their predicted molecular weights using anti-His, anti-HA, and anti-Strep-II tag antibodies (Figure 2b–d).

### 3.2. Qualitative and Quantitative Analysis of Ergothioneine

**Figure 3:**
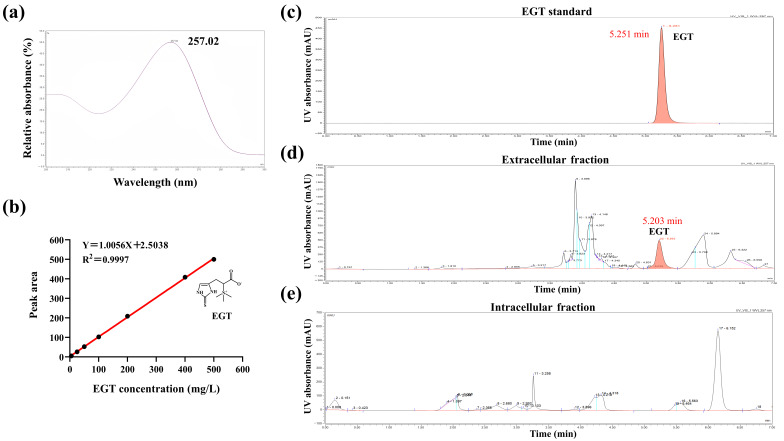
Establishment of the HPLC analytical method and chromatographic analysis of EGT. (**a**) UV absorption spectrum of the EGT standard indicating a maximum absorption peak at 257 nm. (**b**) Standard calibration curve for EGT quantification. (**c**–**e**) HPLC chromatograms of (**c**) the EGT standard, (**d**) the extracellular fraction of the engineered strain E2, and (**e**) the intracellular fraction of the engineered strain E2.

In this study, the EGT standard was subjected to a full-wavelength scan (200–400 nm) using high-performance liquid chromatography with diode array detection (HPLC-DAD), which revealed a prominent characteristic absorption peak at 257 nm (Figure 3a). Precision was evaluated using standard solutions spanning 5 to 500 mg/L [72]; the relative standard deviation (RSD) of the EGT response values ranged from 1.17% to 4.28% across all tested concentrations (Table 3). Linear regression analysis, based on the mean peak area (Y) versus EGT concentration (X), demonstrated a strong linear relationship within the 5–500 mg/L range. Fitted by the least-squares method, the regression equation was Y = 1.0056X + 2.5038, with a correlation coefficient (R^2^) of 0.9997 (Figure 3b).

**Figure 4:**
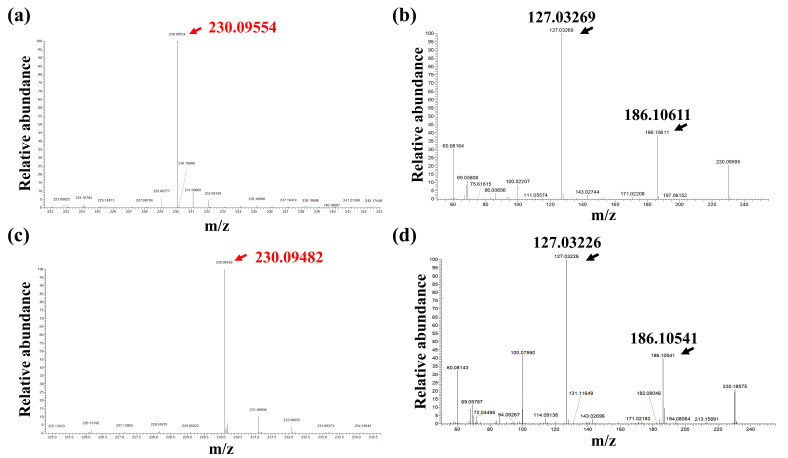
High-resolution mass spectrometry (HRMS) identification of EGT. (**a**,**c**) MS1 spectra and (**b**,**d**) MS2 spectra of the EGT standard and the engineered strain sample. (**a**,**b**) represent the standard, while (**c**,**d**) represent the fermentation sample.

The fermentation products of the engineered strain E2 were analyzed by HPLC. The EGT standard exhibited a single characteristic absorption peak at 257 nm with a retention time of 5.251 min (Figure 3c). In the fermentation supernatant, a distinct peak was detected at 5.203 min (Figure 3d), consistent with the standard. In contrast, no corresponding EGT signal was observed in intracellular samples (Figure 3e). Subsequently, mass spectrometry analysis showed that the theoretical [M + H]^+^ *m*/*z* of the EGT standard was 230.09554 (Figure 4a), whereas the sample exhibited an *m*/*z* of 230.09482 (Figure 4c), indicating high agreement. Following collision-induced dissociation (CID) of the precursor ion, both standard and sample produced identical characteristic fragment ions, with prominent peaks at *m*/*z* 127.03 and 186.100 (Figure 4b,d).

### 3.3. Fermentation Kinetics and Amino Acid Supplementation Analysis of the Engineered Strain

**Figure 5:**
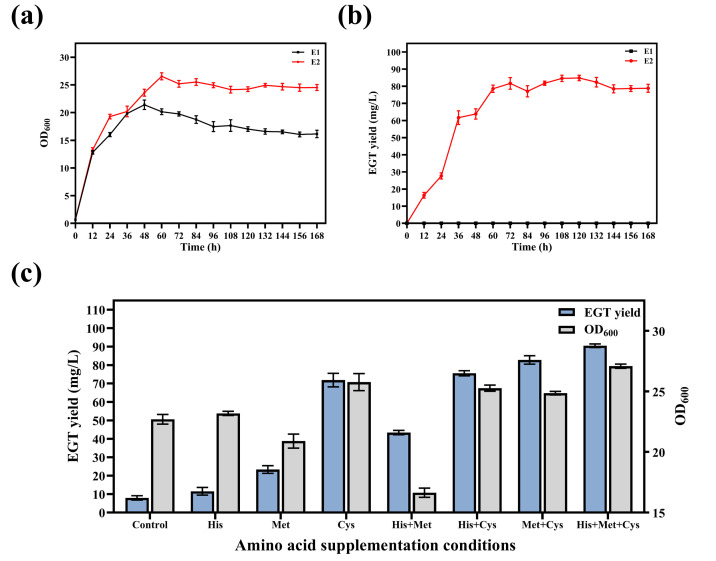
Fermentation kinetics and optimization of precursor amino acid supplementation. (**a**) Growth curves (OD_600_) of the control strain E1 and the engineered strain E2 during 168 h of fermentation. (**b**) Comparison of EGT yield between strains E1 and E2 over the 168 h time course. (**c**) Effects of single and combined supplementation of His, Met, and Cys on EGT yield and biomass (OD_600_) at 120 h.

To investigate the impact of the heterologous EGT biosynthetic pathway on host growth and its accumulation patterns, the fermentation performance of the control strain E1 and the engineered strain E2 was compared. The results showed that both E1 and E2 were in a rapid logarithmic growth phase during the early stage of fermentation, with E2 exhibiting a higher growth rate compared to E1. E2 reached its maximum biomass at approximately 60 h, with an OD_600_ of 26.55 ± 0.65, and maintained a high level from 72 h to 168 h. In contrast, E1 peaked at 48 h with an OD_600_ of 21.42 ± 0.85, followed by a gradual decline (Figure 5a). No EGT synthesis was detected in E1 throughout the fermentation process. For E2, significant product formation was observed 12 h after induction, entering a rapid production phase between 24 h and 60 h. The yield increased progressively, peaking at 120 h and reaching 84.84 ± 1.64 mg/L, with no significant further increase thereafter (Figure 5b).

To further explore the production potential of the engineered strain E2 and identify key metabolic bottlenecks in the biosynthetic pathway, the effects of supplementing precursor amino acids on EGT yield and cell growth were investigated with a blank control containing no exogenous amino acids. Among the single-addition groups, Cys exhibited the most significant impact on yield enhancement. Upon Cys supplementation, the EGT yield increased from 7.97 ± 1.19 mg/L in the control group to 71.81 ± 3.69 mg/L. Dual-precursor strategies further improved yields: the combinations His + Cys and Met + Cys resulted in yields of 75.52 ± 1.39 mg/L and 82.75 ± 2.29 mg/L, respectively. When His, Met, and Cys were supplemented simultaneously, the EGT yield rose to 90.48 ± 0.95 mg/L, representing an approximately 10.35-fold increase compared to the control group. Notably, the triple-supplementation group also supported the highest biomass, with an OD_600_ of 27.08 ± 0.18 (Figure 5c).

### 3.4. Optimization of Induction Strategies

To balance the expression intensity of the heterologous metabolic pathway with the metabolic capacity of the host cell and to achieve the soluble expression of key enzymes in the EGT biosynthetic pathway for high-yield production, the effects of induction temperature (25 °C, 30 °C, and 37 °C) and IPTG concentration (0.1 mM, 0.2 mM, and 0.4 mM) on cell physiological state, target protein expression, and EGT yield were investigated.

**Figure 6:**
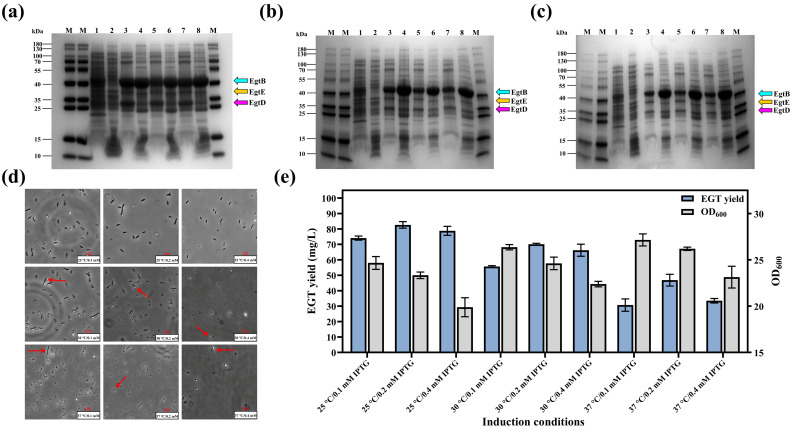
Optimization of induction strategy for EGT production in the engineered strain E2. (**a**–**c**) SDS-PAGE analysis of protein expression at (**a**) 25 °C, (**b**) 30 °C, and (**c**) 37 °C. Lane M: Protein molecular weight marker; Lanes 1, 3, 5, and 7 represent the supernatant fractions, while Lanes 2, 4, 6, and 8 correspond to the pellet fractions of control strain E1 (Lanes 1–2) and engineering strain E2 induced with 0.1 mM (Lanes 3–4), 0.2 mM (Lanes 5–6), and 0.4 mM (Lanes 7–8) IPTG. (**d**) Microscopic observation of cell morphology; red arrows indicate the formation of inclusion bodies. (**e**) Effects of induction temperature and IPTG concentration on EGT yield and biomass (OD_600_).

The results showed that obvious inclusion bodies were formed at 30 °C and 37 °C, whereas no significant inclusion bodies were observed at 25 °C. Comparing the 0.1 mM and 0.4 mM IPTG groups, the biomass density remained moderate under 0.2 mM IPTG induction (Figure 6d). SDS-PAGE analysis revealed that at 25 °C, distinct target bands were present in both the soluble (supernatant) and insoluble (pellet) fractions. At 30 °C, the target protein bands in the pellet intensified, while those in the supernatant became fainter. At 37 °C, the target proteins were predominantly localized in the pellet, with much lower expression levels observed in the supernatant (Figure 6a–c). Fermentation results indicated that EGT yields remained high across all groups induced at 25 °C. In contrast, although the high temperature (37 °C) favored rapid biomass accumulation, it resulted in the lowest EGT yield. Regarding the IPTG concentration, the EGT yield peaked at 82.63 ± 2.11 mg/L under the 25 °C/0.2 mM condition, with a corresponding OD_600_ of 23.33 ± 0.35. However, further increasing the IPTG concentration to 0.4 mM led to a decrease in OD_600_ to 19.88 ± 1.03 and a reduction in yield to 78.80 ± 2.95 mg/L (Figure 6e).

### 3.5. Optimization of Solubility-Enhancing Tags

**Figure 7:**
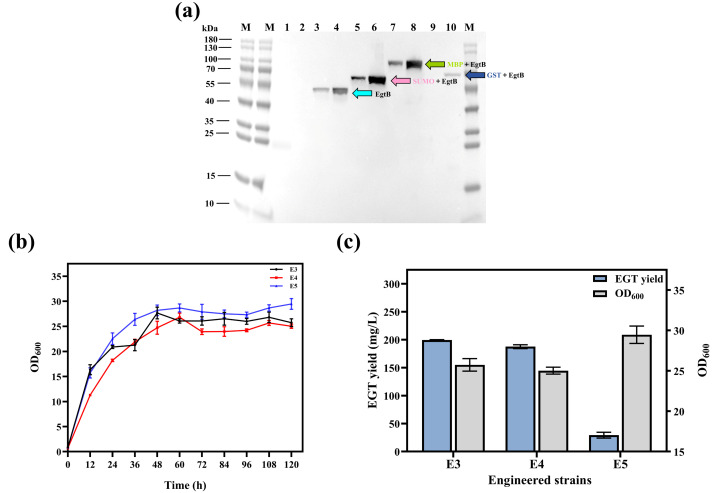
Screening and evaluation of solubility-enhancing tags for EgtB expression. (**a**) Western blot analysis of EgtB fusion proteins. Lane M: Protein molecular weight marker; Lanes 1, 3, 5, 7, and 9 represent the supernatant fractions; Lanes 2, 4, 6, 8, and 10 represent the pellet fractions. Lanes 1–2: EgtB (control strain E1); Lanes 3–4: EgtB (strain E2); Lanes 5–6: SUMO-EgtB (strain E3); Lanes 7–8: MBP-EgtB (strain E4); Lanes 9–10: GST-EgtB (strain E5). (**b**) Growth kinetics of engineered strains. The biomass (OD_600_) of strains E3 (SUMO-tagged), E4 (MBP-tagged), and E5 (GST-tagged) was monitored over a 120 h fermentation period. (**c**) Comparison of EGT yield and final biomass. EGT yield and biomass (OD_600_) were measured after 120 h of cultivation for strains E3, E4, and E5.

After validation via colony PCR (Supplementary Figure S3) and sequencing (Supplementary Figure S5), the recombinant plasmids were transformed into *E. coli* BL21 (DE3) to construct strains E3, E4, and E5. Following IPTG induction, Western blot analysis was conducted on the supernatant and pellet fractions of E3, E4, and E5. The theoretical molecular weights of the fusion proteins are listed in Table 2. Compared to strain E2, the supernatants of the SUMO- and MBP-fusion strains (E3 and E4) displayed markedly stronger signals corresponding to the target proteins. Conversely, almost no target signals were detected in the supernatant of the GST group, with the target proteins predominantly localized in the insoluble pellet fraction (Figure 7a).

Analysis of the growth profiles revealed that all three strains underwent rapid proliferation within the initial 48 h of fermentation before transitioning into the stationary phase. Strain E5 demonstrated the most vigorous growth, attaining a maximum OD_600_ of 29.47 ± 1.08 at 120 h, which was significantly higher than that of strains E3 and E4 (Figure 7b). Regarding EGT production, the performance of the different tags varied significantly. Strain E3 achieved the highest EGT yield, reaching 199.42 ± 0.64 mg/L, followed by E4 with a yield of 187.65 ± 3.45 mg/L. Notably, despite exhibiting the highest OD_600,_ strain E5 produced the lowest EGT yield of only 29.31 ± 5.24 mg/L (Figure 7c).

### 3.6. Rational Design and Analysis of Reinforced Pathways for Key Precursor Supply

**Figure 8:**
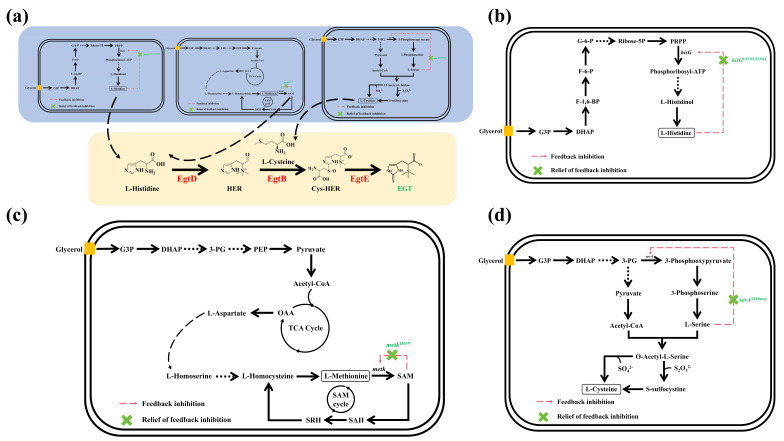
Rational design of precursor metabolic networks and strategies for relieving feedback inhibition. (**a**) Global biosynthetic scheme for ergothioneine production, integrating the optimized pathways for His, SAM, and Cys. (**b**) Schematic diagram of His biosynthesis and metabolic regulation. (**c**) Schematic diagram of SAM biosynthesis and metabolic regulation. (**d**) Schematic diagram of Cys biosynthesis and metabolic regulation.

**Figure 9:**
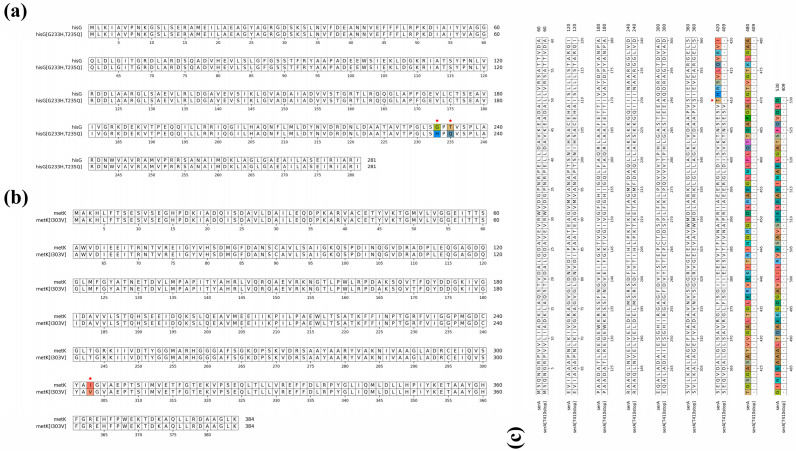
Amino acid sequence alignment of wild-type and corresponding mutant proteins (**a**) Sequence alignment of HisG with HisG^G233H,T235Q^. (**b**) Sequence alignment of MetK with MetK^I303V^. (**c**) Sequence alignment of SerA with SerA^T410stop^. Red stars indicate the successful introduction of targeted mutations or truncation sites.

The precursor metabolic network was systematically engineered to construct multiple pathways capable of relieving feedback inhibition (Figure 8a). First, in the histidine biosynthetic pathway, ATP phosphoribosyltransferase (HisG, encoded by the *hisG* gene) is a key rate-limiting enzyme that is subject to feedback inhibition by histidine [73]. To address this, site-directed mutagenesis was performed on *hisG* to introduce G233H and T235Q double mutations (Figure 8b). Amino acid sequence alignment confirmed the accuracy of the mutation sites (Figure 9a). Second, S-adenosylmethionine (SAM) serves as the methyl donor for the histidine methylation reaction. SAM is synthesized by Methionine Adenosyltransferase (MAT, encoded by the *metK* gene); however, high concentrations of SAM exert strong feedback inhibition on this enzyme [74]. Therefore, an I303V mutation was introduced into *metK* via site-directed mutagenesis (Figure 8c), and the accuracy of the mutation was confirmed by sequence alignment (Figure 9b). Finally, cysteine serves as the sulfur donor for EGT synthesis, and its biosynthesis is highly dependent on the accumulation of the upstream precursor, serine. D-3-phosphoglycerate dehydrogenase (SerA, encoded by the *serA* gene) is susceptible to feedback inhibition by serine, thereby restricting the metabolic flux toward cysteine [75]. Consequently, a premature stop mutation was introduced at the 410th amino acid position of *serA* (Figure 8d). Amino acid sequence alignment confirmed the successful introduction of this mutation (Figure 9c).

The mutants *hisG*^G233H,T235Q^, *metK*^I303V^, and *serA*^T410stop^ were individually cloned into the low-copy vector pCDFDuet-1. After validation via colony PCR (Supplementary Figure S4) and sequencing (Supplementary Figure S5), the recombinant plasmids were transformed into E3 competent cells. The engineered strains E7, E8, and E9 were obtained through dual-antibiotic selection (kanamycin and streptomycin) and verified by colony PCR (Supplementary Figure S4). Subsequently, combinatorial constructs of the mutant genes were prepared and cloned into the pCDFDuet-1 vector. Following verification by sequencing (Supplementary Figure S5), the recombinant plasmids were transformed into E3 competent cells. The engineered strains E10, E11, E12, and E13 were obtained through dual-antibiotic selection (kanamycin and streptomycin) and further verified via colony PCR (Supplementary Figure S4).

**Figure 10:**
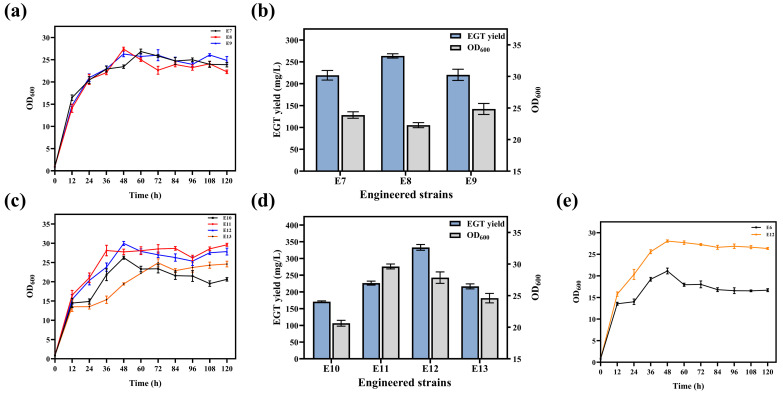
Impact of metabolic pathway optimizations on EGT production and cell growth. (**a**) Growth curves and (**b**) EGT yield and biomass (OD_600_) of strains E7, E8, and E9. (**c**) Growth curves and (**d**) EGT yield and biomass (OD_600_) of strains E10, E11, E12, and E13. (**e**) Comparison of growth kinetics between the optimized strain E12 and the control strain E6.

Growth curves revealed that strains E7, E8, and E9 entered the stationary phase at approximately 48 h. The final OD_600_ of E8 was 22.28 ± 0.41, which was slightly lower than those of E7 and E9 (Figure 10a). Regarding EGT production, E8 exhibited the most superior performance, achieving a peak yield of 263.61 ± 4.95 mg/L (Figure 10b). Further analysis showed that E11 displayed the most robust growth capacity, reaching an OD_600_ of 29.62 ± 0.39 after 120 h (Figure 10c). However, strain E12 demonstrated the optimal production performance, with an EGT yield reaching 333.20 ± 8.67 mg/L (Figure 10d). Finally, to evaluate the impact of the multi-level engineering strategy on the physiological state of the host cells, the growth kinetics of the optimized strain E12 were compared with those of the control strain E6. Both strains rapidly entered the logarithmic phase upon inoculation and reached peak biomass at 48 h. E12 achieved a maximum OD_600_ of 28.07 ± 0.28, which was significantly higher than that of E6 (21.13 ± 0.68). In the late fermentation stage, the control strain E6 exhibited a marked decline in OD_600_, eventually dropping to 16.7 ± 0.35, whereas E12 maintained high stability, with an OD_600_ of 26.35 ± 0.22 at 120 h. Overall, E12 demonstrated superior growth and metabolic stability compared to E6 (Figure 10e).

### 3.7. Screening of Carbon Sources and Optimization of Precursor Supplementation

**Figure 11:**
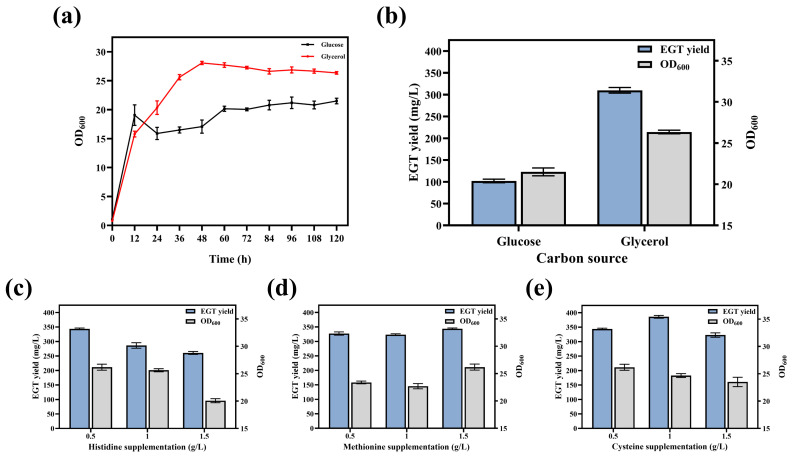
Impact of carbon source and precursor supplementation on EGT production and cell growth. (**a**) Growth curves and (**b**) EGT yield and biomass (OD_600_) of strain E12 using glucose or glycerol as the carbon source. (**c**–**e**) Effects of varying supplementation concentrations of (**c**) Histidine, (**d**) Methionine, and (**e**) Cysteine on EGT yield and biomass (OD_600_).

To identify the optimal carbon source for efficient EGT accumulation, the impacts of glucose and glycerol on the growth performance and EGT biosynthetic capacity of strain E12 were investigated and compared. Results showed that while the glucose-supplemented group (20 g/L) exhibited rapid initial growth during the first 12 h post-inoculation, it subsequently entered a distinct growth stagnation phase. The OD_600_ values in this group fluctuated significantly and remained below 23.00 throughout the fermentation. In contrast, the glycerol-supplemented group (5.04 g/L) demonstrated superior growth robustness. The biomass increased steadily after inoculation, peaking at an OD_600_ of approximately 28.07 ± 0.28 at 48 h, and remained at a high level (above 26.00) during the late fermentation stage (Figure 11a). After 120 h of fermentation, the EGT yield in the glucose group was only 101.98 ± 4.19 mg/L, whereas the glycerol group achieved a significantly higher yield of 310.02 ± 6.47 mg/L, representing a 2.04-fold increase over the glucose group (Figure 11b).

Although a triple-precursor supplementation strategy was previously established, the supply levels of these precursors not only affect the growth of the chassis cells but also directly determine the EGT biosynthetic capacity of strain E12. Therefore, an optimal precursor supply scheme was determined to achieve efficient EGT accumulation. The results showed that the biomass of the strain remained at a high level when the histidine concentration was 0.5 g/L or 1.0 g/L. However, when the concentration was increased to 1.5 g/L, cell growth was significantly inhibited, and the EGT yield showed a downward trend. The highest EGT yield, reaching 343.62 ± 2.72 mg/L, was obtained with 0.5 g/L histidine (Figure 11c). Further investigation revealed that both the OD_600_ and EGT yield reached their maximum values when the methionine concentration was set at 1.5 g/L (Figure 11d). Finally, the evaluation of cysteine supplementation showed that the yield increased with the concentration within the range of 0.5–1.0 g/L. At 1.0 g/L cysteine, the EGT yield peaked at 385.70 ± 4.86 mg/L. However, when the concentration was further increased to 1.5 g/L, both the OD_600_ and EGT yield decreased (Figure 11e).

## 4. Discussion

The heterologous EGT biosynthetic system developed in this study shows both alignment and divergence from previously reported strategies, particularly regarding production yield, fermentation duration, and engineering approaches. In shake-flask cultivation, the optimal engineered strain constructed in this study achieved a higher final EGT titer in comparison with previously reported *Escherichia coli* and *Saccharomyces cerevisiae* systems. However, it should be noted that the fermentation period employed in the present work was longer, resulting in a lower volumetric productivity relative to the earlier *Escherichia coli* study [51,76]. To address these limitations, future research will focus on integrating fermentation scale-up with the metabolic optimization of the chassis cell, thereby further shortening the fermentation cycle and fully unlocking the production potential of the engineered strain. Regarding engineering strategies, most existing studies rely on model microorganisms with well-characterized genetic backgrounds. In this study, *E. coli* was selected as the chassis for the heterologous expression of the *egtB* gene from *Methylobacterium pseudosasicola*, alongside the *egtD* and *egtE* genes from *Mycobacterium smegmatis*. Compared to the traditional five-step pathway, this approach reduces the metabolic burden imposed by the heterologous genes and effectively circumvents competition for precursors with the host’s endogenous glutathione biosynthetic pathway [77,78].

A major limitation in the heterologous synthesis of EGT in *E. coli* lies in the low soluble expression of key rate-limiting enzymes. In this study, EgtB, identified as the primary rate-limiting enzyme, exhibited poor solubility and a strong propensity to form inclusion bodies, consistent with previous observations of Osawa et al. [79]. To improve the soluble expression of EgtB, several solubility-enhancing tags, including SUMO, MBP, and GST tags, were introduced [80]. Among them, fusion with the SUMO tag not only significantly enhanced the soluble expression of EgtB but also resulted in the highest EGT production, indicating that increasing the solubility of rate-limiting enzymes is a critical strategy for strengthening EGT biosynthetic capacity in *E. coli* [81]. In this study, despite its high solubility, the GST tag failed to assist in the correct folding of EGT biosynthetic enzymes. As previously demonstrated by Kapust and Waugh [82], the GST tag appears to lack typical chaperone-like properties, such as deep hydrophobic clefts. Accordingly, it may not effectively sequester and shield folding intermediates, potentially leading to the rapid aggregation of the enzymes into inactive inclusion bodies. It is hypothesized that the inactivation of these key enzymes blocks metabolic flux through the heterologous pathway, thereby alleviating the metabolic burden on the chassis cells and allowing for the redirection of energy toward proliferation. Ultimately, this represents a possible explanation for the observed contrasting phenotype of high biomass and low product yield. Another critical bottleneck is the limited supply of essential precursors. Histidine, methionine, and cysteine serve as key substrates for EGT synthesis, and their insufficient supply restricts heterologous production in *E. coli* [50]. To assess production potential and identify metabolic constraints, the effects of individual precursor supplementation were systematically evaluated. The results revealed distinct differences in their contributions, with cysteine showing the most significant effect. Combinations of dual precursors that included cysteine exhibited synergistic effects, whereas the simultaneous addition of histidine, methionine, and cysteine resulted in the maximal EGT yield. This finding is in agreement with Yan et al. [53], confirming that the coordinated supply of sulfur donors, methyl donors, and histidine is essential for efficient EGT biosynthesis. To further enhance metabolic flux toward these core precursors, rational engineering of key genes (*hisG*^G233H,T235Q^, *metK*^I303V^, and *serA*^T410stop^) [83,84,85] was performed to relieve feedback inhibition. The double-mutant strain E12 (*metK*^I303V^-*serA*^T410stop^) exhibited superior production performance. Notably, strain E13, incorporating all three mutations, yielded less EGT than E12, confirming that in metabolic engineering, more mutation sites do not necessarily correlate with higher efficiency. Excessive modification may impose a severe metabolic burden or disturb precursor balance, ultimately impairing cell growth and product formation [86,87]. Efficient EGT synthesis further requires a sufficient and balanced supply of the three precursor amino acids. Previous reports indicate that histidine biosynthesis is metabolically costly, consuming substantial intracellular ATP, whereas high concentrations of cysteine exhibit cytotoxicity. Conversely, sufficient methionine provides methyl donors by sustaining the SAM cycle [88,89,90]. By optimizing precursor supplementation to 0.5 g/L of histidine, 1.5 g/L of methionine, and 1.0 g/L of cysteine, the metabolic load was balanced with cell growth, leading to a final EGT yield of 385.70 ± 4.86 mg/L.

To optimize the fermentation process, a standard fermentation duration of 120 h was determined based on the observed trends in biomass and product accumulation. The ideal induction conditions were identified as 25 °C and 0.2 mM IPTG, which effectively promoted the soluble expression of key enzymes, minimized inclusion body formation, and maximized the balance between cell growth and product synthesis. These findings are consistent with previous reports suggesting that low temperatures and moderate IPTG concentrations are widely recognized as effective strategies for enhancing protein solubility and EGT yield in engineered strains [52]. Additionally, glycerol was found to be a significantly superior carbon source compared to glucose, which is consistent with the conclusion reported by Yu et al. [91]. It is hypothesized that this superiority stems from the ability of glycerol to mitigate acetate accumulation and carbon catabolite repression [92]. Moreover, glycerol metabolism likely provides more abundant reducing power and energy compared to glucose, thereby creating a more favorable metabolic environment for the efficient biosynthesis of EGT [93,94].

In this study, the synthesized EGT was found to be primarily secreted into the fermentation supernatant, with no significant intracellular accumulation detected. This finding is consistent with the results reported by Tanaka et al. [44], confirming the efficient EGT efflux capacity of the engineered strain. Such superior secretory capacity may greatly facilitate the simplification of downstream procedures, including product extraction, separation, and purification [95]. Remarkably, the optimized engineered strain exhibited markedly improved growth performance relative to the control strain. It is hypothesized that this growth improvement arises from several synergistic physiological benefits. Possible explanations include the potential cytoprotective role of endogenously synthesized EGT in relieving intracellular oxidative stress, as well as the reduced accumulation of toxic metabolic byproducts enabled by the efficient efflux mechanism [96,97,98]. However, the precise underlying mechanisms warrant further investigation in future studies.

## 5. Conclusions

In this study, *E*. *coli* BL21 (DE3) was used as the chassis to successfully construct a heterologous biosynthesis system for ergothioneine. By employing strategies such as heterologous gene assembly, soluble modification of key enzymes, reinforcement of precursor metabolism, and optimization of the fermentation process, the expression of rate-limiting enzymes and the efficiency of precursor supply were effectively improved. Consequently, the EGT synthesis performance of the engineered strain was significantly enhanced. Specifically, the *egtB* gene from *Methylobacterium pseudosasicola* was combined with the *egtD* and *egtE* genes from *Mycobacterium smegmatis* to construct a simplified three-step catalytic pathway for EGT synthesis. The soluble expression of the rate-limiting enzyme EgtB was enhanced via fusion with a SUMO tag. Furthermore, feedback inhibition in precursor synthesis was relieved through targeted mutations at key sites of the *hisG*, *metK*, and *serA* genes. Coupled with the synergistic optimization of the carbon source, induction temperature, IPTG concentration, and precursor amino acid supplementation, the EGT production capacity of the strain was remarkably increased. However, the overall production efficiency still requires further improvement. Future studies can focus on systematic optimizations, including enzyme engineering, the reconstruction of precursor metabolic networks, and the regulation of fed-batch fermentation, to further enhance the potential of *E. coli* as a sustainable platform for EGT biosynthesis.

## Figures and Tables

**Table 1:** Plasmids and strains used in this study.

Names	Description	Sources
Plasmids		
pRSFDuet-1	Expression vector, RSF replicon, Kan resistance	Lab stock
pCDFDuet-1	Expression vector, CloDF13 replicon, Sm resistance	Lab stock
pRSF-*egtBDE*	pRSFDuet-1 containing *egtB*, *egtD*, *egtE*	This work
pRSF-*SUMO-egtBDE*	pRSFDuet-1 containing *SUMO*, *egtB*, *egtD*, *egtE*	This work
pRSF-*MBP-egtBDE*	pRSFDuet-1 containing *MBP*, *egtB*, *egtD*, *egtE*	This work
pRSF-*GST-egtBDE*	pRSFDuet-1 containing *GST*, *egtB*, *egtD*, *egtE*	This work
pCDF-*hisG*^G233H,T235Q^	pCDFDuet-1 containing *hisG*^G233H,T235Q^	This work
pCDF-*metK*^I303V^	pCDFDuet-1 containing *metK*^I303V^	This work
pCDF-*serA*^T410stop^	pCDFDuet-1 containing *serA*^T410stop^	This work
pCDF-*hisG*^G233H,T235Q^-*metK*^I303V^	pCDFDuet-1 containing *hisG*^G233H,T235Q^, *metK*^I303V^	This work
pCDF-*hisG*^G233H,T235Q^-*serA*^T410stop^	pCDFDuet-1 containing *hisG*^G233H,T235Q^, *serA*^T410stop^	This work
pCDF-*metK*^I303V^-*serA*^T410stop^	pCDFDuet-1 containing *metK*^I303V^, *serA*^T410stop^	This work
pCDF-*hisG*^G233H,T235Q^-*metK*^I303V^-*serA*^T410stop^	pCDFDuet-1 containing *hisG*^G233H,T235Q^, *metK*^I303V^, *serA*^T410stop^	This work
Strains		
*E. coli* DH5α	Cloning strain	Lab stock
*E. coli* BL21 (DE3)	Expression strain	Lab stock
E1	*E. coli* BL21 (DE3) harboring pRSFDuet-1	This work
E2	*E. coli* BL21 (DE3) harboring pRSF-*egtBDE*	This work
E3	*E. coli* BL21 (DE3) harboring pRSF-*SUMO*-*egtBDE*	This work
E4	*E. coli* BL21 (DE3) harboring pRSF-*MBP*-*egtBDE*	This work
E5	*E. coli* BL21 (DE3) harboring pRSF-*GST*-*egtBDE*	This work
E6	*E. coli* BL21 (DE3) harboring pRSFDuet-1 and pCDFDuet-1	This work
E7	*E. coli* BL21 (DE3) harboring pRSF-*SUMO*-*egtBDE* and pCDF-*hisG*^G233H,T235Q^	This work
E8	*E. coli* BL21 (DE3) harboring pRSF-*SUMO*-*egtBDE* and pCDF-*metK*^I303V^	This work
E9	*E. coli* BL21 (DE3) harboring pRSF-*SUMO*-*egtBDE* and pCDF-*serA*^T410stop^	This work
E10	*E. coli* BL21 (DE3) harboring pRSF-*SUMO*-*egtBDE* and pCDF-*hisG*^G233H,T235Q^-*metK*^I303V^	This work
E11	*E. coli* BL21 (DE3) harboring pRSF-*SUMO*-*egtBDE* and pCDF-*hisG*^G233H,T235Q^-*serA*^T410stop^	This work
E12	*E. coli* BL21 (DE3) harboring pRSF-*SUMO*-*egtBDE* and pCDF-*metK*^I303V^-*serA*^T410stop^	This work
E13	*E. coli* BL21 (DE3) harboring pRSF-*SUMO*-*egtBDE* and pCDF-*hisG*^G233H,T235Q^-*metK*^I303V^-*serA*^T410stop^	This work

**Table 2:** Theoretical molecular weights and expected total molecular weights of recombinant proteins.

Target Protein	Theoretical MW (kDa)	Fusion Tag	Expected Total MW (kDa)
EgtB	47.3	His	48.8
EgtD	35.0	Strep-II	36.7
EgtE	39.0	HA	40.7
His-EgtB	48.8	SUMO	60.2
His-EgtB	48.8	MBP	89.4
His-EgtB	48.8	GST	74.5

**Table 3:** Precision evaluation of the HPLC method for EGT determination.

EGT Standard Concentration (mg/L)	Peak Area			Mean (n = 3)	RSD/%
5	5.0394	5.1620	5.2245	5.1420	1.83
25	25.9105	26.4536	25.9395	26.1012	1.17
50	51.0290	51.9651	55.3279	52.7740	4.28
100	102.9408	104.7449	101.0592	102.9150	1.79
200	209.5304	212.6862	204.5879	208.9348	1.95
400	412.1293	414.5872	399.1243	408.6136	2.03
500	508.4368	505.9857	486.2346	500.2190	2.43

## Data Availability

The original contributions presented in this study are included in the article/Supplementary Material. Further inquiries can be directed to the corresponding authors.

## Supplementary Materials

The following supporting information can be downloaded at: https://www.mdpi.com/article/10.3390/microorganisms14051088/s1. Table S1: Primers used in this study; Figure S1: Confirmation of *egtB, egtD, egtE*, by colony PCR. Figure S2: Restriction enzyme digestion analysis of recombinant plasmid pRSF-*egtBDE*. Figure S3: Confirmation of *SUMO*, *MBP*, *GST*, by colony PCR. Figure S4: PCR verification for the construction of recombinant strains E7–E13. Figure S5: Sequence validation of the recombinant plasmids.
