# Supplementary material for: Engineering Escherichia coli for Ergothioneine Production via Metabolic Engineering and Fermentation Optimization
Source: Microorganisms. 2026 May 11;14(5):1088. doi: 10.3390/microorganisms14051088 (PMC13209761; doi:10.3390/microorganisms14051088)
Supplement: Supplementary file 1 [file microorganisms-14-01088-s001.zip › Supplementary Material.pdf]

Supplementary Material

Supplementary Table S1. Primers used in this study.

Primer name	Sequence (5′–3′)
egtB-F	ATGGCGGCGACCGCAGCGCT
egtB-R	TTATGCTACATCAGCCAGGCGTAGGCCGG
egtD-F	ATGACCCTGAGTCTGGCAAACTATC
egtD-R	TTACCTAACGGCCAGCGAC
*egt*E-F	ATGATGTTGGCACAGCAGTGG
egtE-R	TTAAGGAGCTTCTCTTAACGCGG
SUMO-F	ATGTCGGACTCAGAAGTCAATC
SUMO-R	TCCACCAATCTGTTCTCTGTGAG
MBP-F	ATGAAAATCGAAGAAGGTAAACTGGT
MBP-R	AGTCTGCGCGTCTTTCAG
GST-F	ATGTCCCCTATACTAGGTTATTGG
GST-R	TTTTGGAGGATGGTCGCCAC
hisG^{G233H,T235Q}-F	ATGCTGAAAATTGCCGTTCC
hisG^{G233H,T235Q}-R	TTAAATGCGAGCAATACGGATTTC
metK^{I303V}-F	ATGGCGAAACATCTGTTCACTAGC
metK^{I303V}-R	TTACTTCAGACCCGCTGCATC
serA^{T410stop}-F	ATGAGTCAGAATGGCCGTC
serA^{T410stop}-R	TTAGACGCTTTCACTGTTCGTTTTC

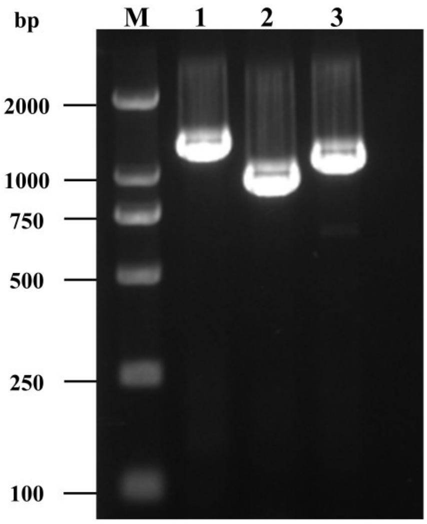

Supplementary Figure S1. Confirmation of *egtB*, *egtD*, *egtE*, by colony PCR. Lane M: DL2000 DNA marker; lanes 1-3: *egtB*, *egtD*, and *egtE* in *E. coli* DH5α (1269 bp, 966 bp, and 1116 bp, respectively), amplified with high-fidelity Taq polymerase.The results show that all amplified bands are highly

consistent with their theoretical sizes.

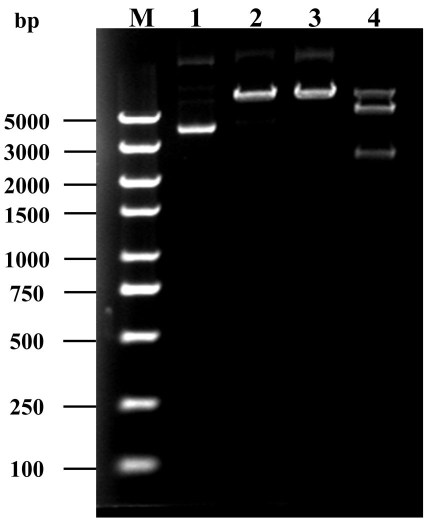

Supplementary Figure S2. Restriction enzyme digestion analysis of recombinant plasmid pRSF-*egtBDE*. Lane M: DL5000 DNA marker; lane 1: uncut plasmid; lane 2: *Hind*III-digested plasmid (7240 bp); lane 3: *Xho*I-digested plasmid (7240 bp); lane 4: *Hind*III/*Xho*I double-digested fragments (4863 bp and 2377 bp). The results show that all amplified bands are highly consistent with their theoretical sizes.

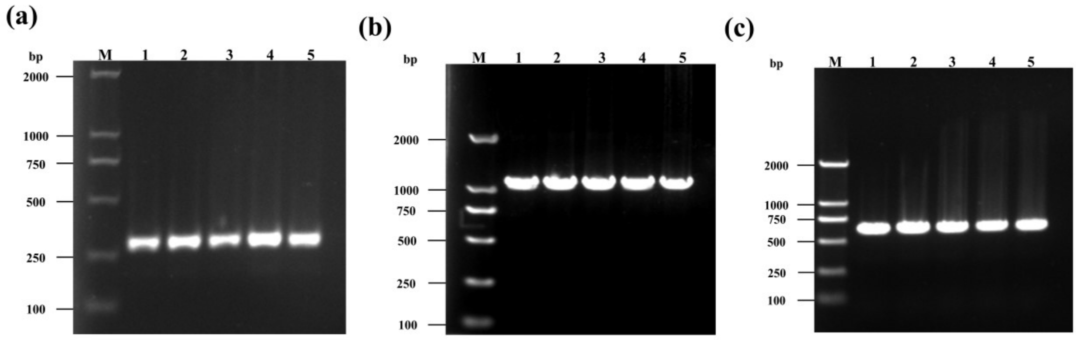

Supplementary Figure S3. Confirmation of *SUMO, MBP, GST,* by colony PCR. Lane M: DL2000 DNA marker; (a), (b), and (c) correspond to strains E3 (SUMO-tag, 294 bp), E4 (MBP-tag, 1101 bp), and E5 (GST-tag, 654 bp), respectively. Lanes 1-5: five independent colonies selected for PCR screening. The results show that all amplified bands are highly consistent with their theoretical sizes.

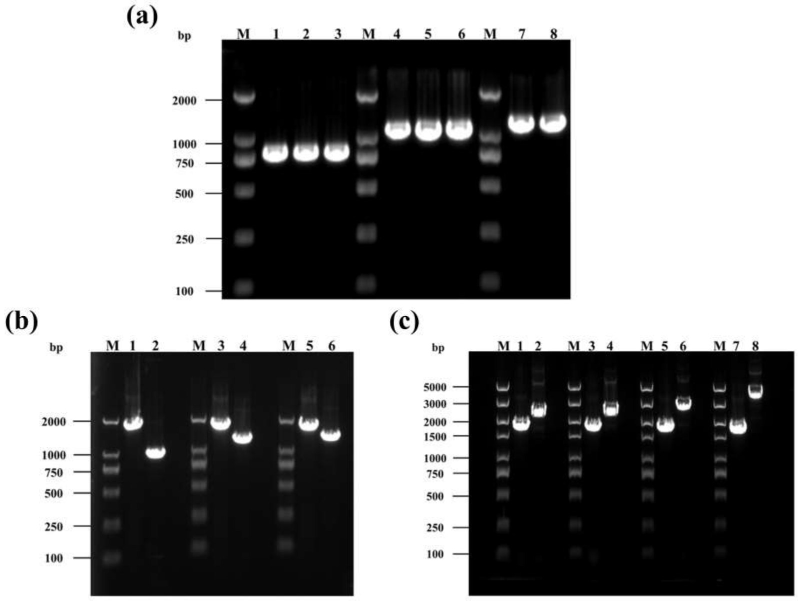

Supplementary Figure S4. PCR verification for the construction of recombinant strains E7-E13. Lane M: DL2000 DNA marker or DL5000 DNA marker; (a) Amplification of mutant gene fragments. lanes 1-3: (*hisG*^{G223H,T235Q}, 846 bp); lanes 4-6: (*metK*^{I303V}, 1155 bp); lanes7-8: (*serA*^{T410stop}, 1230 bp). (b, c) Colony PCR verification of strains (b) E7-E9 and (c) E10-E13. Lanes 1, 3, 5, and 7 represent the verification of the first plasmid pRSF-*SUMO-BDE*; Lanes 2, 4, 6, and 8 correspond to the newly introduced mutant genes or their respective combinations. All amplified fragments align with their expected molecular weights, confirming the successful construction of the dual-plasmid recombinant strains.

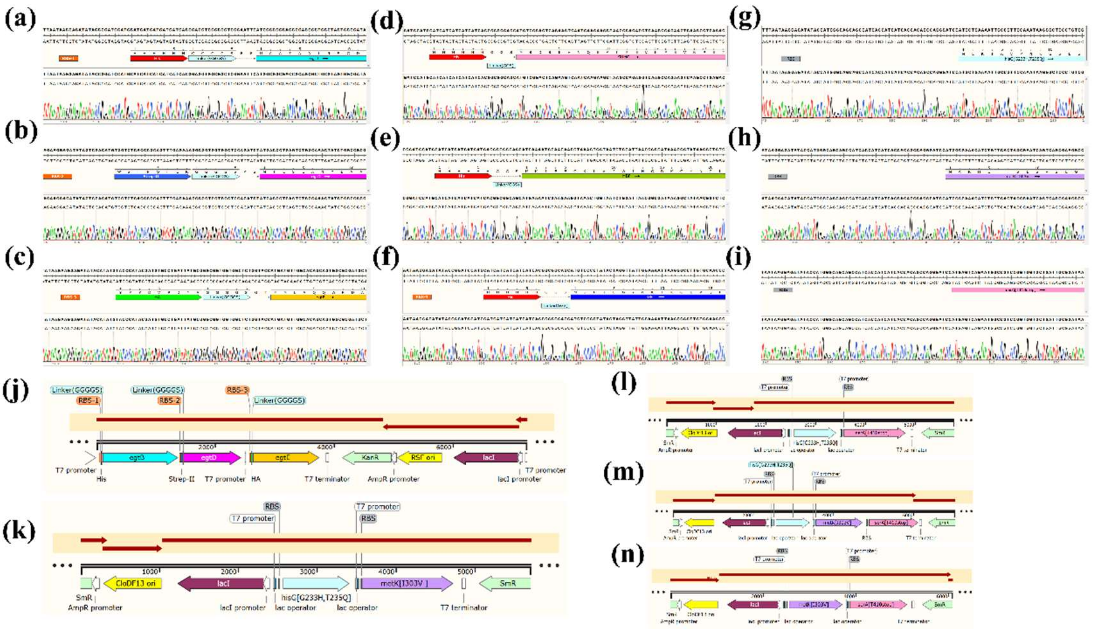

Supplementary Figure S5. Sequence validation of the recombinant plasmids. (a-i) Sanger sequencing chromatograms of *egtB*, *egtD*, *egtE* genes, fusion tags (His, Strep-II, and HA), and mutant genes (*hisG*^{G233H,T235Q}, *metK*^{I303V}, and *serA*^{T410stop}). The raw sequencing chromatogram signals are clear, and the results are completely consistent with the target sequences. (j-n) Third-generation whole-plasmid sequencing

results: (j) map of the pRSFDuet-*egtBDE* recombinant plasmid; (k-n) sequencing maps of recombinant plasmids with different mutant gene combinations . All sequencing results are perfectly aligned with the target sequences.
